# Management of myogenic ptosis in chronic progressive external ophtalmoplegia

**Published:** 2014-07-04

**Authors:** Mohammad Taher Rajabi, Seyed Ziaeddin Tabatabaie, Mohammad Bagher Rajabi, Yalda Abrishami, Seyedeh Simindokht Hosseini, James Oestreicher

**Affiliations:** 1 Department of Ophthalmology, School of Medicine, Eye Research Center, Farabi Eye Hospital, Tehran University of Medical Sciences, Tehran, Iran; 2 Department of Ophthalmology and Vision Sciences, School of Medicine, University of Toronto, Toronto, Canada

**Keywords:** Myogenic Ptosis, Chronic Progressive External Ophthalmoplegia, Levator Resection, Sling, Lower Lid Recession, Spacer Graft

## Introduction

Patients with mitochondrial myopathies, of which chronic progressive external ophthalmoplegia (CPEO) is the most common, can present with ptosis and proximal limb weakness.^1^ Ptosis associated with CPEO can provide challenging management problems. This form of ptosis may have poor levator function. Additional features must be considered in determining the surgical treatment. For example, these patients may have limited upgaze and a poor Bell’s phenomenon, poor orbicularis function, dry eye, and resultant poor corneal protective mechanisms.^2^ This would put them at greater risk for exposure keratopathy after ptosis repair. In this article, we describe our experience with a patient with CPEO who had pre-operative moderate levator function and good orbicularis function that after performing levator resection developed severe exposure keratopathy.

**Figure 1:**
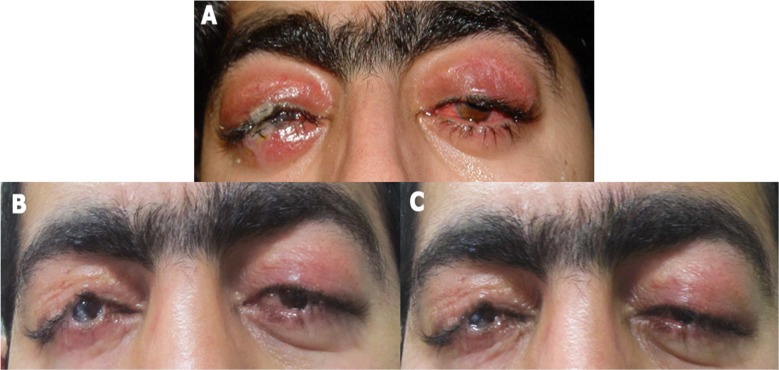
(A) Photograph showing eyelid edema and corneal injection 2 months after the first surgery in right tarsorrhaphied eye. (B) Photograph 5 years after the first surgery showing narrow palpebral fissure in both eye with corneal opacity at the right eye. (C) Corneal exposure during eyelid closure. Exposure at both eyes is visible even after closure in tarsorrhaphied eyes.

A 20-year-old man was referred to Farabi Eye Hospital, Tehran, Iran with bilateral lagophthalmos. He had a Hutchinson myopatic face similar to the CPEO facial pattern. He had lid swelling. Visual acuity was 2/10 in both eyes. Slit lamp examination revealed corneal edema, epithelial defects bilaterally, and signs of corneal exposure keratopathy (Figure 1A).

The patient had been undergone bilateral levator resection 2 weeks before in a city at North of Iran. His pre-operative visual acuity was 10/10 and bilateral ptosis was present with levator function measuring 8-mm in both eyes. The histological report showed ragged red fibers compatible with CPEO.

To avoid more severe complications from corneal exposure, tarsorrhaphy was performed on both eyes. After 3 weeks and when corneal signs subsided, one of the tarsorrhaphied eyes was opened, but after 10 days, he represented with signs of exposure keratopathy and again underwent tarsorrhaphy. Tarsorrhaphy and opening of the sutured lids was repeated 4 times in a period of 5 months, and then due to corneal haziness and vascularization the surgery was revised. Both upper eyelids were so tight and without any movement so that after opening of tarsorrhaphy, upper lid retracted and there was not any option unless repeating tarsorrhaphy.

In both eyes after opening of the previous incision, the levator was recessed. Lower lid recession with posterior lamellar scleral spacer graft was performed on both eyes for narrowing the vertical palpebral fissure. After this operation, he had mild ptosis with marginal reflex distance measuring 2 mm, and lower lid elevation, so that the vertical palpebral fissure measured 5 mm. Slit lamp examination showed corneal haziness and vascularization in both eyes. Acuity decreased in the right eye to 1/10 and left eye to 4/10. No worsening of signs and symptoms were noticed during the 60 months follow-up (Figure 1B and C). However, due to extension of corneal vascularization and thinning the right eye, vision was 3 m finger count in both eyes.

In patients with CPEO and ptosis, even with cautious approaches, lagophthalmos with corneal exposure will usually ensue, partially due to increased vertical palpebral fissure and partially due to poor orbicularis function, in addition to poor corneal protective mechanisms.^2^

To overcome such complication Shorr et al.^3^ have advocated upper eyelid surgery in conjunction with lower eyelid elevation to improve visually significant ptosis and to maintain corneal protection in these patients. In this procedure, the ptosis repair was combined with maximum recession of the lower eyelid, using a posterior lamellar scleral spacer graft. This combined procedure allows a more aggressive upper lid elevation while minimizing the overall change in vertical palpebral fissure. The overall pre-operative vertical palpebral fissure height is maintained or even decreased. Thus, a more significant improvement in superior visual field is obtained with maintenance of eyelid closure and without the accompanying exposure risk.^3^

In patients with moderate to poor levator function, between 4 and 8 mm levator resection has been recommended. However, attention just to levator function in determining the surgical treatment is misleading. Since preoperative good function of orbicularis in these patients is not exactly equivalent to its’ function postoperatively, and because of underlying problems such as poor Bell’s phenomenon and poor corneal protection, it is recommended not to increase palpebral fissure height. If one performs levator resection it is better to recess the lower lid with a spacer graft to maintain a narrow, vertical interpalpebral fissure to maximize eyelid closure ability, and reposition this opening centrally over the pupil. One can argue that because the disease is progressive, and sling surgery is adjustable and potentially reversible (by removal), this technique is preferable to levator resection surgery.^4^

This article suggests that ptosis surgery in CPEO be performed only when vision is compromised by lid height and affects the visual axis. Surgery should be done with full follow-up care. We recommend lower lid recession, using a spacer graft to be combined with sling (with silicone) or levator resection procedures, in poor or good levator function, respectively. This procedure is done to avoid accompanying exposure keratopathy due to increased palpebral fissure and to allow eyelid closure.

## Notes

**How to cite this article:** Rajabi MT, Tabatabaie SZ, Rajabi MB, Abrishami Y, Hosseini SS, Oestreicher J. Management of myogenic ptosis in chronic progressive external ophtalmoplegia. Iran J Neurol 2014; 13(3): 185-7.
